# An opiine Braconidae (Hymenoptera) reared from Richardiidae (Diptera) and recognition of a new species group of *Opius* s. l.

**DOI:** 10.3897/zookeys.289.4900

**Published:** 2013-04-12

**Authors:** Robert Wharton, Sophia Daniels, Xanthe Shirley, Danielle Restuccia

**Affiliations:** 1 Department of Entomology, Texas A&M University, College Station, Texas 77843 U.S.A.

**Keywords:** Parasitoid, fly, *Sepsisoma*, HAO, Poaceae

## Abstract

A new species of opiine Braconidae, *Opius rojam* Daniels & Wharton, is described from Trinidad. The description is based in part on two individuals reared from *Sepsisoma erythrocephalum* infesting shoots of the grass *Paspalum fasciculatum*. This is the first record of members of the Opiinae attacking species in the dipteran family Richardiidae. The *Opius ingenticornis* species group is proposed and delineated to accommodate this and several putatively related species formerly included in *Opius* (*Merotrachys*), *Opius* (*Pendopius*), and *Opius* (*Ilicopius*). A key to the species of this group is provided. Descriptions and diagnoses are referenced to the Hymenoptera Anatomy Ontology.

## Introduction

Members of the braconid subfamily Opiinae develop as koinobiont endoparasitoids of various cyclorrhaphous Diptera, emerging from the puparium of their hosts. This biological trait defines Opiinae + Alysiinae relative to all other Braconidae. The most commonly recorded hosts of Opiinae belong to the families Agromyzidae, Tephritidae, and Anthomyiidae, at least in part because these families harbor a large number of economically important species. Hosts belonging to 11 additional families of cyclorrhaphous Diptera have also been recorded. These include Calliphoridae, Chloropidae, Diopsidae, Drosophilidae, Ephydridae, Lonchaeidae, Muscidae, Phoridae, Psilidae, Scathophagidae, and one species of Tachinidae (Yu et al. 2012), though some of these need to be vetted. The primary purpose of this contribution is to record a new family of hosts for Opiinae: the Richardiidae.

Fischer (1972, 1977, 1987) monographed the Opiinae on a world basis and numerous changes in the classification have subsequently been published. There are at least 1981 valid species in the Opiinae (Yu et al. 2012) and 116 genus group names (84 of these currently treated as valid by one or more authors) have been applied to various combinations of these species (Wharton et al. 2012). There are over 50 subgenera now in use (Yu et al. 2012), the majority of these assigned to *Opius* Wesmael s. l. Van Achterberg and Salvo (1997) restricted the name *Opius* to species with a basal lobe on the mandible, referring to a classification in press that has yet to be published. Until a more complete classification is offered, we prefer to treat *Opius* more broadly as a repository for the bulk of the Opiinae whose relationships remain uncertain, largely following the approach of Fischer (1972) and Wharton (1997a, b), as explained more recently in Wharton et al. (2012). The species treated here belong in *Opius* s. l. sensu Fischer (1972, 1999) and Wharton (1997a, b) or in *Phaedrotoma* Foerster sensu Van Achterberg and Salvo (1997). A key to genera that provides delineation of *Phaedrotoma* in this sense was recently published by Li et al. (2013). The characterization of the new species group described here and its placement within the current classification is a secondary goal of the study.

## Materials and methods

**Specimens**. Reared material from Trinidad was kindly sent for study to the senior author by Paul Marsh (formerly USDA Systematic Laboratory, Washington, D. C.). Other specimens used in this study, primarily consisting of primary type material of previously described species, were borrowed from or examined at the following institutions: American Entomological Institute, Gainesville, Florida, USA (AEIC), California Academy of Sciences, San Francisco, California, USA (CAS), Canadian National Collection, Ottawa, Ontario, Canada (CNC), Naturhistorisches Museum Wien, Vienna, Austria (NHNW), Texas A&M University Insect Collection, College Station, Texas, USA (TAMU), and the U. S. National Museum of Natural History, Washington, D. C., USA (USNM).

In the material examined section under each species description, we record the data label for holotypes exactly as these data appear on the labels. We use a more standardized format for labels on paratypes, labels on additional specimens examined, and for data published on other specimens. Images of label data can be found at http://mx.speciesfile.org/projects/8/public/otu_group/show/386.

**Figures**.Images were acquired digitally using either Helicon Focus® or Syncroscopy’s AutoMontage® software, mostly in combination with either a ProgRes 3008 or a Zeiss AxioCam MRc5 digital camera mounted on a Leica MZ APO dissecting microscope. A few images were also acquired with a Leica M205C equipped with an internal camera. All images were further processed using various minor adjustment levels in Adobe Photoshop® such as image cropping and rotation, adjustment of contrast and brightness levels, color saturation, and background enhancement. Compiled images, including many not incorporated here, are available in color and high resolution at http://mx.speciesfile.org/projects/8/public/otu_group/show/386.

**Database management, digital dissemination, and ontology reference**.Illustrations and free-text diagnoses for morphospecies were assembled in mx, a web-based content management system that facilitates data management and dissemination for taxonomic and phylogenetic works (e.g. Yoder et al. 2006). The mx project is open source, with code and further documentation available at http://sourceforge.net/projects/mx-database/. Data pertinent to this work, including images, diagnoses, and descriptions, are available at http://mx.speciesfile.org/projects/8/public/otu_group/show/386. The multiple entry key found at this site was also generated in mx.

Morphological terms used in this revision were matched to the Hymenoptera Anatomy Ontology (HAO, Yoder et al. 2010, Seltmann et al. 2012). Identifiers (URIs) in the format http://purl.obolibrary.org/obo/HAO_XXXXXXX represent anatomical concepts in HAO version http://purl.obolibrary.org/obo/hao/2011-05-18/hao.owl, as used by Wharton et al. (2012, Appendix). The URIs are provided to enable readers to confirm their understanding of the anatomical structures being referenced. To find out more about a given structure, including images, references, and other metadata, use the identifier as a web-link, or use the HAO:XXXXXXX (note colon replaces underscore) as a search term at http://glossary.hymao.org. Terminology as linked through the HAO (Wharton et al. 2012 Appendix) largely follows Sharkey and Wharton (1997), with a few additions from Walker and Wharton (2011) and Wharton et al. (2012). Measurements largely follow Walker and Wharton (2011) except where indicated.

## Results

**Biology.** Two individuals representing a previously undescribed species very similar to *Opius ingenticornis* Fischer were reared by Fred Bennett from individually isolated puparia of *Sepsisoma erythrocephalum* (Schiner) (Diptera: Richardiidae) in Curepe, St. George Co., Trinidad. A nearly circular emergence hole, with jagged edges typical of many opiines, is located near the anterior end of each of the two puparia: dorsally on one puparium and ventrally on the other. Richardiid biology is generally poorly known, with a few records for species in other genera developing in flowers or rotting vegetation (Hancock 2010). The fly larvae from which these wasps were reared were collected from shoots of the grass *Paspalum fasciculatum* Wild. ex Fluegge (Poaceae), that were exhibiting deadheart (Deeming 1985). The wasps that emerged from these puparia are described below as *Opius rojam* Daniels & Wharton, sp. n. See also the remarks section under *Opius ingenticornis* below.

There are no host records for any of the other members of the *ingenticornis* species group. The relative rarity of biological information on richardiids may explain this, and we therefore predict that most if not all of the members of this species group may eventually be found to utilize richardiids as hosts.

**Figures 1–4.**
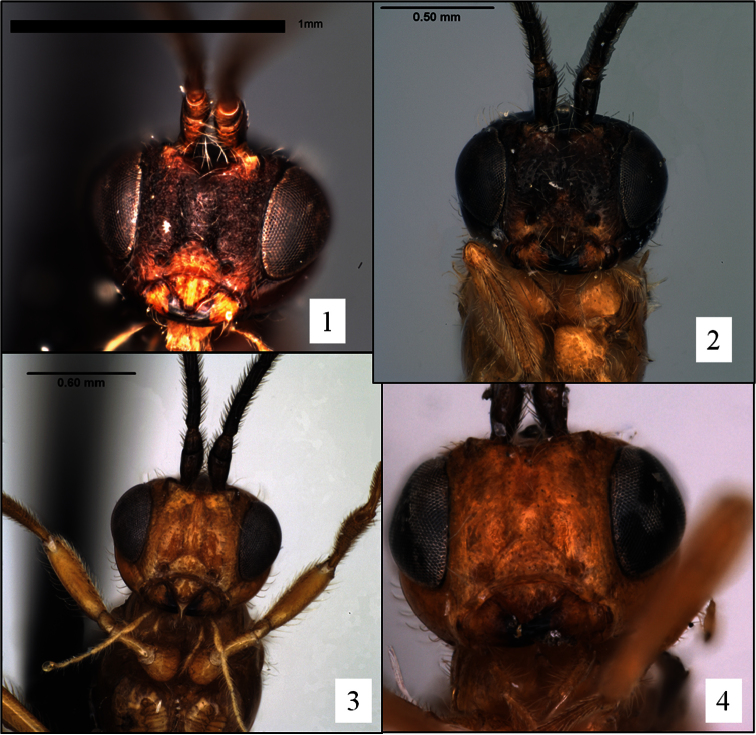
*Opius* spp., face. **1** *Opius matthaei* Fischer, holotype, showing granular sculpture **2** *Opius raphaeli* Fischer, holotype **3** *Opius melchioricus* Fischer **4** *Opius rojam* Daniels & Wharton sp. n., holotype.

**Figures 5–8.**
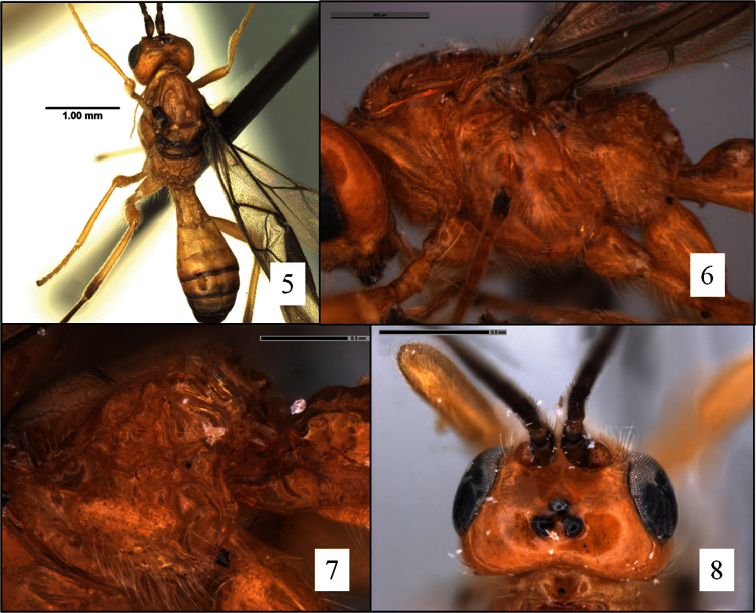
*Opius* spp. **5** *Opius melchioricus* Fischer, mesosoma, dorsal view**6** *Opius rojam* Daniels & Wharton sp. n., holotype, mesopleuron **7** *Opius rojam* holotype, propodeum posterior-lateral view **8** *Opius rojam* head and pronotum, dorsal view, showing enlarged pronope.

**Figures 9–12.**
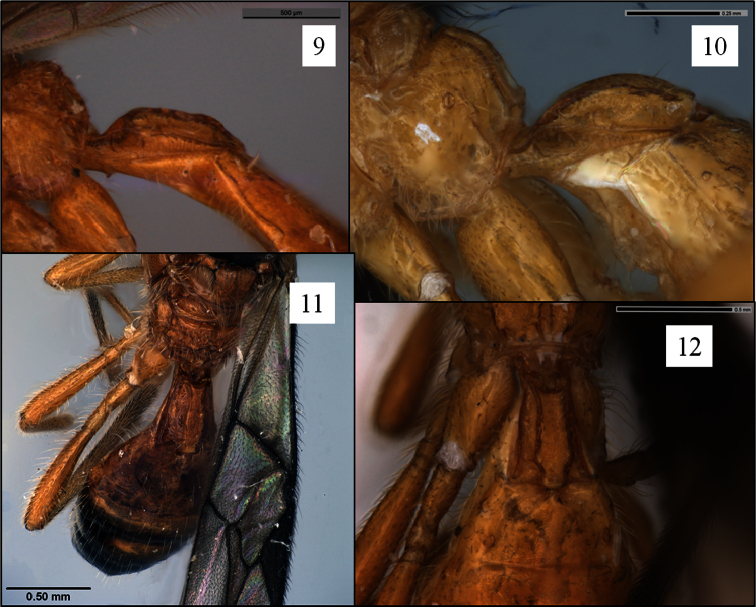
*Opius* spp. holotypes, petiole (T1). **9** *Opius rojam* Daniels & Wharton sp. n., lateral view **10** *Opius bicarinifer* Fischer, lateral view **11** *Opius raphaeli* Fischer, dorsal view **12** *Opius curiosicornis* Fischer, dorsal view.

**Figures 13–16.**
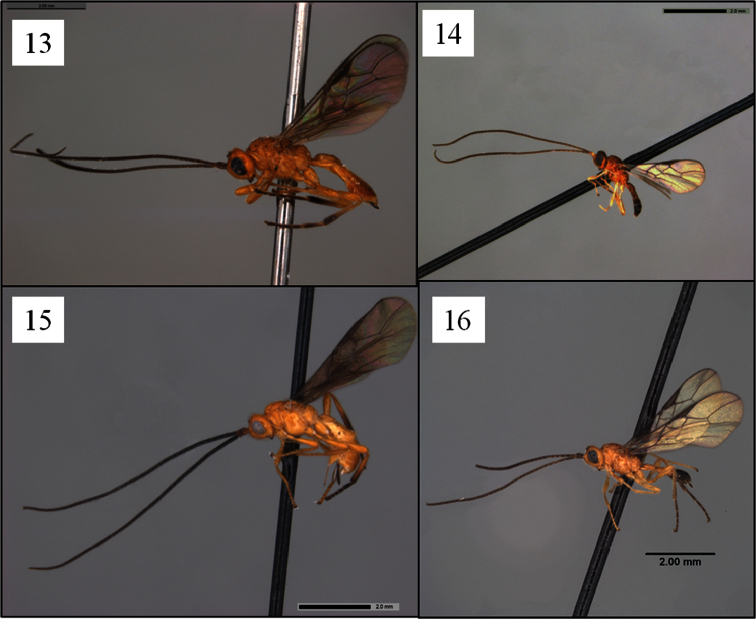
*Opius* spp. holotypes, habitus. **13** *Opius rojam* Daniels & Wharton sp. n. **14** *Opius antennatus* Fischer **15** *Opius curiosicornis* Fischer **16** *Opius gabrieli* Fischer.

**Figures 17–20.**
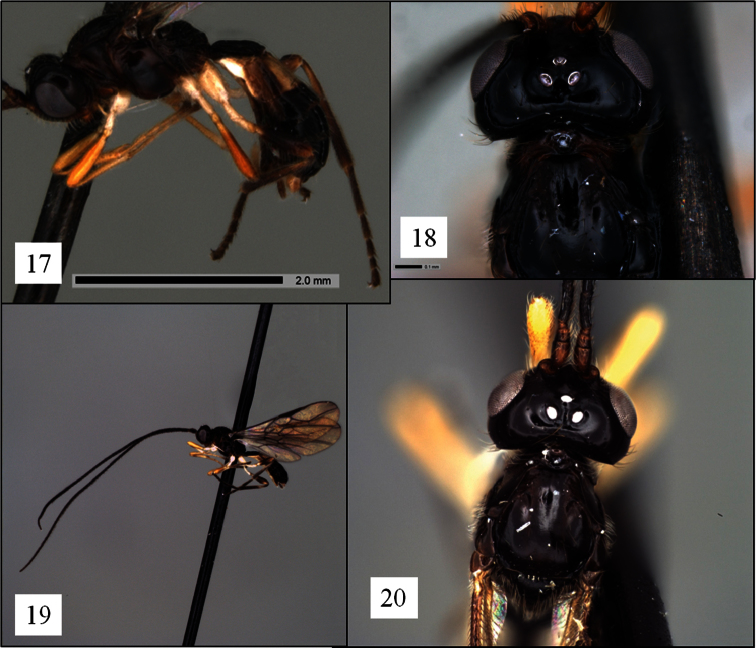
*Opius* spp., holotypes **17** *Opius albericus* Fischer, habitus **18** *Opius albericus* head in dorsal view **19** *Opius pilosicornis* Fischer, habitus **20** *Opius pilosicornis* head in dorsal view.

**Genus group placement and characterization.** *Opius rojam* and *Opius ingenticornis* belong to an exclusively New World group of species that hereafter will be referred to as the *ingenticornis* species group. Among species of *Opius* s. l. with a distinctly exposed labrum (Figs 1–4) but lacking a mesoscutal midpit (Fig. 5), a precoxal sulcus (Fig. 6, 17, 33), and a basal lobe on the mandible (Figs 1–4), they are most readily recognized by the combination of very long antennae (Figs 13–16; known species with 45–62 flagellomeres), huge pronope (Figs 5, 8), and the relatively distinctive petiole (Figs 9, 11–12). Most of the species treated here were placed by Fischer (1977, 1979a) in the subgenus *Merotrachys* Fischer, with a few also in the subgenus *Pendopius* Fischer. Subsequent changes in the subgeneric classification, summarized in Fischer (1999), would necessitate the transfer of some of these species to *Ilicopius* Fischer, though this has never been formally done. In the classification suggested by van Achterberg and Salvo (1997), these species would all be placed in the genus *Phaedrotoma*. The species treated here were selected based on an examination of nearly all of the primary types of *Ilicopius*, *Merotrachys*, *Opius* (*Opius*) sensu Fischer (1977), and *Pendopius*. It is possible, however, that we have overlooked other described species that should be placed in the *ingenticornis* species group.

**Figures 21–24.**
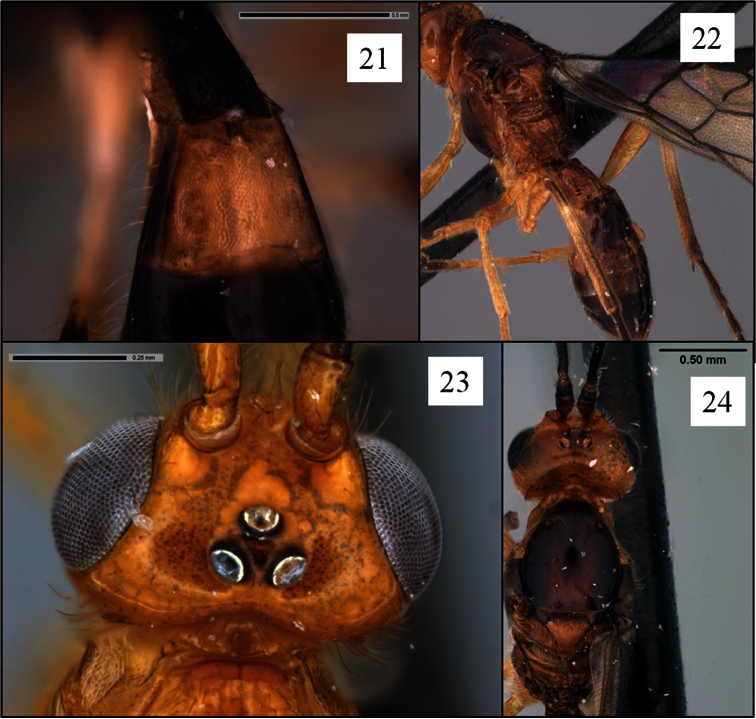
*Opius* spp. holotypes. **21** *Opius albericus* Fischer, T2+3 sculpture **22** *Opius michaeli* Fischer, T2+3 sculpture **23** *Opius bicarinifer* Fischer, head in dorsal view **24** *Opius michaeli*, head in dorsal view.

Fischer (1972) initially characterized *Pendopius* on the basis of reduced body sculpture relative to *Merotrachys* but subsequently (Fischer 1999), he restricted *Pendopius* to species with the maxillary palp much longer than head height. The maxillary palp is difficult to measure on intact specimens, which is problematic since about half of the species treated below are known only from the holotype. Nevertheless, though variable within the *ingenticornis* species group (Figs 2–3), none of the species has the palp sufficiently long to be placed in *Pendopius* sensu Fischer (1999). T2+3 is shagreened in many of these species (Fig. 21), leading to their placement in *Merotrachys*. Those without sculpture on T2+3 fall within *Ilicopius* based on Fischer (1999). Unfortunately, the sculpture exhibits a gradient from extensive and readily visible to patchy and virtually absent across species and also among specimens within a species, greatly reducing the diagnostic value of this character state. Thus, the species that we include in the *ingenticornis* species group fall into at least two and possibly even three subgenera within Fischer’s (1972, 1999) classification of *Opius* s. l. Since these species are notably different from the type species of *Ilicopius*, *Merotrachys*, and *Pendopius*, and yet appear to form a natural group (as delineated below), we have elected to treat them as a species group within *Opius* s. l. but without assigning them to a specific subgenus.

**Figures 25–28.**
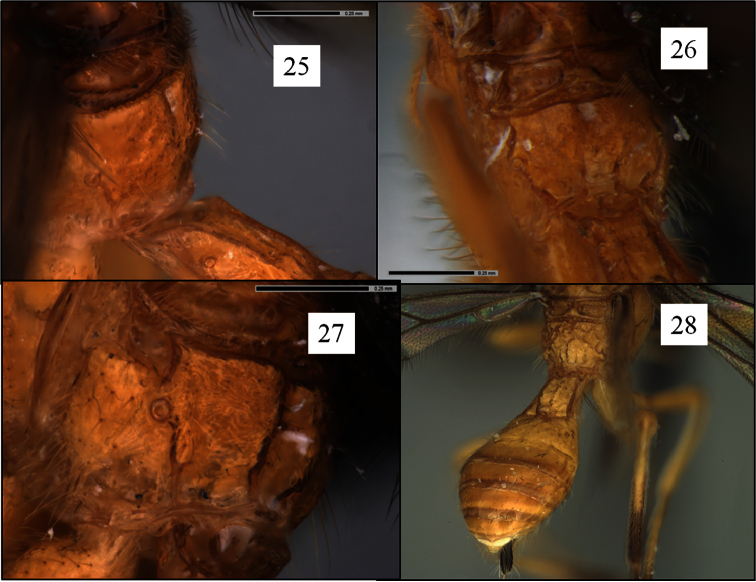
*Opius* spp. holotypes, propodea. **25** *Opius nimifactus* Fischer **26** *Opius macrocornis* Fischer **27** *Opius curiosicornis* Fischer **28** *Opius ingenticornis* Fischer.

**Figures 29–32.**
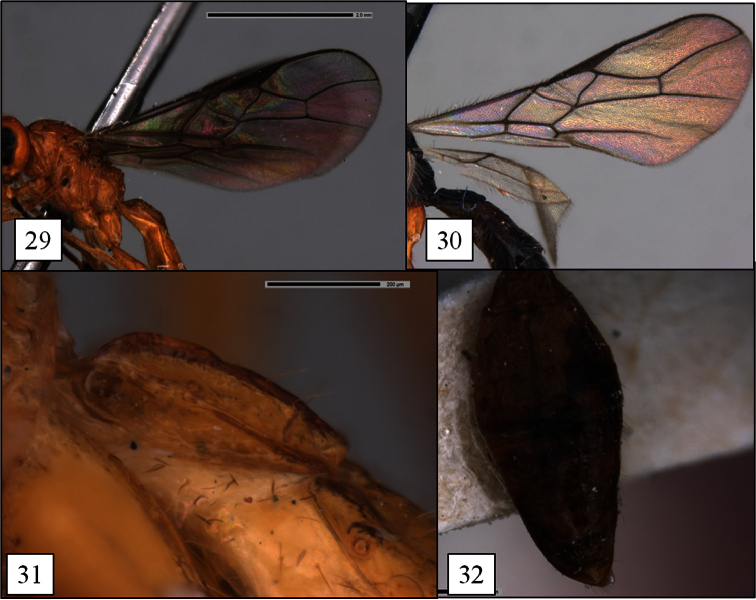
*Opius* spp., holotypes. **29** *Opius rojam* Daniels & Wharton sp. n., fore and hind wing **30** *Opius matthaei* fore wing**31** *Opius curiosicornis* Fischer, T1 lateral view **32** *Opius filiflagellatus* Fischer, metasoma.

The *ingenticornis* species group can be defined as follows: Mandible (Figs 1–4) short, broadly triangular, dorsal margin strongly angled ventrally, broadly exposing labrum. Clypeus (Figs 1–4) shaped as a broad crescent, nearly hemispherical, flat to weakly protruding ventrally, ventral margin shallowly concave, rarely appearing truncate. Malar sulcus distinct, complete. Antenna unusually long (Figs 13–16), approximately twice longer than body; first flagellomere slender, longer than second, with long, narrow plate sensilla. Occipital carina broadly absent dorsally (Figs 8, 18, 20, 24), the gap in dorsal view at least as wide as distance between eyes; carina well developed laterally and ventrally, widely separated from hypostomal carina ventrally. Pronope (Figs 8, 24) deep, wide, posterior margin at least weakly overlapping base of mesoscutum (Fig. 6), thus obliterating posterior transverse sulcus medially; vertical carina absent on pronotum laterally. Mesoscutum (Fig. 5, 24) without midpit; notaulus short, curved, pit-like anteriorly, narrowing and evanescent posteriorly; anterior declivity (Fig. 6) shallow to absent or nearly so. Propodeum (Figs 7, 25–28) with median depression at least anteriorly, never with median longitudinal carina. Mesopleuron (Fig. 6) without sternaulus, precoxal sulcus unsculptured, absent or very faintly indicated; hind margin of mesopleuron not obviously crenulate on dorsal 0.5. Fore wing (Figs 29–30) with second submarginal cell long to very long, 3RSa at least 1.2 × longer than 2RS; m-cu variable: antefurcal, interstitial, or postfurcal relative to 2RS; 2CUb arising from or near middle of first subdiscal cell. Hind wing (Fig. 29) with RS distinctly infumate; m-cu absent. T1 (Figs 9, 11–12) with dorsal carinae parallel or nearly so, extending from base to apex; laterope large, deep; dorsope absent. Sculpture on T2+3 variable, shagreened when present.

The petiole (T1) is notably different from that of the type species of *Ilicopius*, *Merotrachys*, and *Pendopius*, all of which lack the distinctive anterior declivity and have more poorly developed dorsal carinae. The antennae are also shorter in these three species (less so in *Opius ilicis* Nixon than in the other two but still with fewer than 30 flagellomeres) and the mesoscutum is sharply declivitous anteriorly. Many of the species of the *ingenticornis* species group are large (body length 3–4 mm) with longer, more slender mesosomas relative to the more typical opiines that attack agromyzid hosts, such as the type species of *Ilicopius*, *Merotrachys*, and *Pendopius*. Perhaps as a result of the larger size, the setae on the mesoscutum seem longer and more erect. The setae are more densely clustered anteriorly then follow along the lines of the notauli in a single row posteriorly.

In a few of the species treated below several individuals were available for examination. In these, the origin of fore wing m-cu relative to 2RS was variable, and in one case, there was variation between the right and left fore wing. This variation creates difficulties for use of existing keys to species of both *Merotrachys* and *Pendopius* (Fischer 1979a, b). *Merotrachys*, in the sense of Fischer (1972, 1977, 1979a, 1999), consists of species with sculpture on the metasomal tergites posterior to T1. Since the type of sculpture differs among species currently assigned to *Merotrachys* (striate in some, shagreened in others, for example), characterization of *Merotrachys* as monophyletic on this basis alone may be difficult. The type of sculpture in the members of the *ingenticornis*, when present, is all the same (shagreened) and is consistent with monophyly of this species group.

**Species treatment.** The species are treated under two sections below, immediately following the dichotomous key. The first section contains the one newly described species, followed in alphabetical order by the 14 described species that we have included in the *ingenticornis* species group. The second section contains four additional species that are similar in some respects, but which are excluded at the present time. All of the excluded species have complete, well-developed, more or less parallel-sided dorsal carinae on T1, and the configuration of the clypeus, labrum, and mandibles is the same as in the *ingenticornis* species group. These may represent basal members of this putative clade, but are excluded therefrom primarily because of differences in the shape of T1, without a distinct anterior declivity and with the basal depression not as clearly delimited posterior-medially. Additionally, the antennae are either broken or have significantly fewer flagellomeres. Rationale for exclusion is included in the remarks section under each of these four species.

**Key to species of the ingenticornis species group.** Fischer (1977, 1979a, b) provides the most recent dichotomous keys to the species of *Merotrachys* and *Pendopius*. The dichotomous key presented here is modified from these. A multiple entry key can be found at http://mx.speciesfile.org/projects/8/public/site/wharton_lab/home.

**Table:** 

1	Propodeum weakly sculptured laterally: smooth to shagreened between propodeal spiracle/pleural carina and margin of median trough (Figs 25–27). Mesopleuron yellow to orange	2
–	Propodeum coarsely sculptured laterally, rugose to carinately rugose on smooth to shagreened background (Figs 7, 28). Mesopleuron varying from yellow to dark brown	4
2 (1)	Tegula and lateral margin of mesoscutum dark brown to black (Fig. 42)	*Opius nimifactus* Fischer
–	Tegula and lateral margin of mesoscutum yellow to orange	3
3 (2)	Median trough of propodeum deep, sharply carinately margined from base to apex with at least some transverse carinae in trough (Fig. 27)	*Opius curiosicornis* Fischer
–	Median trough relatively shallow, less distinctly margined (Fig. 26)	*Opius macrocornis* Fischer
4 (1)	Head dark brown to black; mesosoma predominantly yellow to orange (Figs 35–36)	5
–	Color pattern not as above, either head pale (Figs 13–16) or, if head dark, then mesosoma also predominantly brown to dark brown (Figs 17, 19)	6
5 (4)	Hind femur, T1, and tegula dark brown to black. T1 and T2+3 distinctly and extensively shagreened (as in Fig. 21)	*Opius petri* Fischer
–	Hind femur, T1, and tegula yellow to pale orange. T1 and T2+3 faintly and sparsely shagreened	*Opius raphaeli* Fischer
6 (4)	Female with ovipositor sheath 0.5–0.6 times length of mesosoma (Fig. 33). Head pale and tegula dark (Fig. 33)	*Opius michaeli* Fischer
–	Male, or female with ovipositor sheath shorter, 0.2–0.4 times length of mesosoma (Figs 13, 15–16). Head dark or pale; if pale, then tegula also pale	7
7 (6)	Head and T1 dark brown to black. Mesosoma predominantly dark (Figs 17, 19, 34)	8
–	Head and T1 predominantly pale: yellow to orange. Mesosoma predominantly pale	10
8 (7)	Hind legs yellow (Fig. 34). Face densely, finely granular (Fig. 1)	*Opius matthaei* Fischer
–	Hind legs white basally, dark brown to black distally (Fig. 17). Face mostly shagreened, partly smooth, polished	9
9 (8)	Head in dorsal view 1.8 times wider than long	*Opius albericus* Fischer
–	Head in dorsal view 2.0 times wider than long	*Opius pilosicornis* Fischer
10 (7)	Vertex and frons infumate (Fig. 46). Nearctic	*Opius antennatus* Fischer
–	Vertex and frons yellow to orange, same color as remainder of head. Neotropical: Brazil, Costa Rica, Peru, Trinidad	11
11 (10)	Tegula dark brown to black; posterior margins of meso- and metathorax dark brown to black (Fig. 5)	*Opius melchioricus* Fischer
–	Tegula yellow to orange; posterior margins of meso- and metathorax yellow to orange	12
12 (11)	Metasomal terga 5 and 6 dark brown to black (Fig. 16)	*Opius gabrieli* Fischer
–	Metasomal terga 5 and 6 yellow to orange	13
13 (12)	T1 predominantly shagreened (Fig. 38)	14
–	T1 more extensively rugose (Fig. 37)	*Opius rojam* Daniels & Wharton, sp. n.
14 (13)	Fore wing with second submarginal cell shorter, 3RSa about 1.2–1.3 times longer than 2RS; m-cu interstitial or weakly postfurcal relative to 2RS	*Opius ingenticornis* Fischer
–	Fore wing with second submarginal cell longer, 3RSa about 1.5 times longer than 2RS; m-cu antefurcal relative to 2RS	*Opius filiflagellatus* Fischer

### The *ingenticornis* species group, included species

#### Opius rojam

Daniels & Wharton sp. n.

urn:lsid:zoobank.org:act:EBE195C9-36B8-43DF-9E44-E15804E0F1B5

http://species-id.net/wiki/Opius_rojam

Figs 4 , 6 - 9 , 13 , 29 , 37, 39–40 , 43

##### Type locality.

Trinidad, St. George Co., Curepe

##### Type material.

Holotype. Female (USNM), first label, first line: Trinidad: St. George second line: Co., Curepe third line: III 1982 fourth line: F.D. Bennett second label, first line: ex puparium second line: Sepsisoma third line: erythroceph- fourth line: alum third label: 82–92

##### Paratypes.

One male, same data as holotype except third label = 82–90 (TAMU). Two females, Costa Rica, Puntarenas Province, Golfito, 25.vi.1976, M. Wasbauer, Malaise trap 8am-5pm (TAMU).

##### Description.

*Female*. Eye in dorsal view 2.1–2.2 × longer than temple, temples not receding; eye in lateral view 2.3–2.5 × longer than temple. Face coarsely shagreened throughout; weakly elevated midridge extending from clypeus to antennal bases bifurcated dorsally by shallow impression extending ventrally from frons; median impression more elongate in Trinidad than in Costa Rica specimens. Clypeus coarsely shagreened; ventral margin concave, strongly impressed, in profile very weakly bulging dorsad impressed ventral margin, otherwise flat; 1.7–1.8 × wider (between anterior tentorial pits) than midheight. Anterior tentorial pit large, diameter 0.3–0.4 × maximum height of clypeus. Malar space 0.7–0.8 × longer than basal width of mandible; malar sulcus deep, marking sharp contrast between shagreened face and smooth, polished gena. Occipital carina broadly absent dorsally, well-developed laterally, widely separately from hypostomal carina ventrally. Mandible broadly triangular, without basal tooth or lobe; dorsal margin reflected ventrally, broadly exposing labrum; with two apical teeth, ventral tooth slightly smaller than and positioned posterior to dorsal tooth. Maxillary palp about as long as height of head. Antenna approximately 1.8 × longer than body, with 55 flagellomeres; first flagellomere 1.1–1.2 × longer than second, 1.25–1.35 × longer than third; first, second, and third flagellomeres 3.1–3.5, 2.6–2.8, and 2.2–2.5 × longer than wide, respectively; setae on basal flagellomeres thin, pale.

Mesosoma 1.5 × longer than high; 2.3 × longer than wide; 1.5–1.6 × higher than wide. Pronope deep, very large, posterior margin flattened, obliterating posterior transverse sulcus and broadly overlapping base of mesoscutum; pronotum laterally with shallow vertical groove lacking carinate anterior margin. Mesoscutum anteriorly on nearly same plane as pronotum, without distinct anterior declivity; with white, weakly decumbent setae around margins and extending in 1–2 rows along traces of notauli to posterior margin, becoming less densely clustered posteriorly; midpit absent. Notaulus deeply impressed as a short, curved line, not extending to anterior margin of mesoscutum, extending posterior-medially nearly to level of anterior margin of tegula; extending laterally towards tegula as groove bordered by very well-developed supramarginal carina. Scuto-scutellar sulcus rectangular, crenulate. Scutellum bare medially, setose laterally. Propodeum coarsely, carinately rugose, with short median trough anteriorly, areola indistinct, largely obscured by sculpture posteriorly; pleural sulcus irregular, mostly obscured by sculpture; propodeal spiracle equidistant from anterior and posterior margins. Mesopleuron smooth, polished, bare except posterior-ventrally; precoxal sulcus not evident in holotype, present in paratypes as short, faintly impressed, unsculptured groove. Metapleuron finely rugulose on ventral 0.5–0.6, evenly covered with long, white setae.

Wings. Fore wing stigma wedge-shaped, discrete distally, approximately 3.6 × longer than wide; r1 shorter than stigma width, arising from basal 0.55 of stigma; 1RS (excluding parastigma) short, 0.15–0.2 × length of 1M; m-cu interstitial; second submarginal cell converging distally, 3RSa 1.15–1.3 × longer than 2RS; 1cu-a usually interstitial with 1M, weakly postfurcal in one female paratype. Hind wing m-cu completely absent; RS and M equally well-developed as pigmented lines.

Metasoma with T1 1.2–1.3 × longer than apical width, apex 1.7–1.9 × wider than base, length 2.9–3.4 × height at spiracle; sharply declivitous anteriorly, with deep, discrete basal depression; surface coarsely rugose; dorsal carinae distinctly elevated, nearly parallel-sided throughout, very weakly diverging posteriorly, not sinuate, transversely carinate between dorsal carinae; laterope large, deep. T2+3 uniformly shagreened, T4 more weakly and irregularly so. Ovipositor short; ovipositor sheath about 0.2–0.3 × length of mesosoma.

Color. Head, body, tegula, fore and mid legs, hind coxa, trochanter, trochantellus, femur, and basal 0.6–0.7 of tibia orange; remainder of hind leg, pretarsi of all legs, antenna, and ovipositor sheath dark brown to black; wings infumate to darkly infumate.

*Male*. Largely as in female with variation as follows: antenna 2.05 × longer than body, with 56 flagellomeres; mesosoma 2.4 × longer than wide; fore wing m-cu postfurcal; T1 with apex 2.0 × wider than base; metasomal tergum and genitalia black.

Body length 3.9–4.0 mm, fore wing length 4.0 mm, mesosoma length 1.45–1.55 mm.

##### Diagnosis.

Face shagreened throughout. Temples in dorsal view not receding. Antenna with 55–56 flagellomeres; setae on basal flagellomeres thin, pale. Mesoscutum anteriorly on nearly same plane as pronotum, without distinct anterior declivity. Propodeum coarsely, carinately rugose, with short median trough anteriorly, areola largely obscured by sculpture posteriorly. Fore wing 3RSa 1.15–1.3 × longer than 2RS. T1 sharply declivitous anteriorly; surface coarsely rugose. T2+T3 distinctly shagreened. Ovipositor short; ovipositor sheath about 0.2–0.3 × length of mesosoma. Head, body, hind coxa and femur orange; antenna without pale subapical ring; wing infumate.

This species is nearly identical to *Opius ingenticornis*, from which it differs primarily in sculpture. Most notably, T1 is extensively shagreened in *Opius ingenticornis* and lacks coarsely rugose sculpture (Fig. 38). In *Opius rojam*, T1 lacks evident shagreening and is coarsely sculptured throughout (Figs 9, 37), including distinct transverse carinae between the dorsal carinae. *Opius rojam* is also a slightly larger species, with somewhat darker wings. For further discussion of related species, see remarks under *Opius gabrieli* below as well as the remarks under *Opius ingenticornis* and *Opius filiflagellatus*.

##### Biology.

The two specimens from Trinidad (holotype and male paratype) were reared from *Sepsisoma erythrocephala* (Diptera: Richardiidae), and associated puparia are pinned with the specimens. Additional details are given above under the Biology heading at the beginning of the results section.

##### Etymology.

This species is dedicated to Major, a dear friend, but for nomenclatural purposes the species name should be treated as an arbitrary combination of letters.

##### Remarks.

The holotype shows evidence of developmental irregularities along the midline of T2+3 (Fig. 40). The antennae are broken in paratypes from Costa Rica, but these specimens otherwise match the reared material from Trinidad. The male and female from Trinidad have approximately the same number of flagellomeres. The flagellomeres are more numerous than in the females of *Opius ingenticornis* but fewer than in the male paratypes of this species as recorded by Fischer (1965c). The apparent difference in antennal length between the male and female of *Opius rojam* from Trinidad may be an artifact since the antennae are strongly curled apically in the female holotype and therefore difficult to measure accurately.

#### Opius albericus

Fischer

http://species-id.net/wiki/Opius_albericus

Figs 17, 18 , 21

Opius (Merotrachys) albericus Fischer, 1979a: 264–267 (key); 267–269 (description).Holotype female in AEIC (examined).
Opius (Merotrachys) albericus : Yu et al. 2005, 2012 (electronic catalogs).

##### Type locality.

Brazil, Rondonia, Vilhena.

##### Type material.

Holotype. Female (AEIC), first label, first line: Vilhena, Rond. second line: XI. ’73 Brazil third line: M. Alvarenga

##### Paratypes.

Two males (not seen), same data as holotype; one male (not seen), Brazil, Mato Grosso, Sinop, 12°31'S, 55°37'W, x.1974, M. Alvarenga.

##### Diagnosis.

Face distinctly punctate, punctures separated by about 1 × their diameter, strongly shagreened adjacent eye margin, otherwise appearing very weakly shagreened to smooth between punctures, though difficult to see because of position on pin. Eye in lateral view about 2.0–2.5 × longer than temple; temples in dorsal view not receding. Antenna of female broken, 42 flagellomeres remaining, male with 52 flagellomeres; setae on basal flagellomeres thick, dark. Mesoscutum anteriorly on nearly same plane as pronotum, without distinct anterior declivity; notaulus extending laterally towards tegula as groove bordered by distinct supramarginal carina. Propodeum coarsely rugose, median areola absent, median trough anteriorly difficult to see but apparently weak, indistinct. Fore wing 3RSa straight, 1.4–1.5 × longer than 2RS; m-cu postfurcal. T1 declivitous anteriorly at about a 45 degree angle, basal pit delimited posterior-medially; surface shagreened throughout; dorsal carinae sinuate, widest subapically, narrowing apically, without obvious transverse carinae between dorsal carinae. T2 uniformly, distinctly shagreened; T3 mostly weakly shagreened, smoother and very finely punctate laterally. Ovipositor short, barely protruding; ovipositor sheath roughly 0.4 × length of mesosoma. Head, mesosoma, T1, T3–T6 dark reddish brown to dark brown; T2 white with narrow, dark brown lateral margins; hind coxa white; hind femur almost completely dark reddish brown; antenna without subapical pale ring; wing lightly infumate.

##### Remarks.

Originally described from the female holotype and 3 male paratypes. This species, described from western Brazil, is nearly identical to *Opius pilosicornis*, described from Peru. Fischer (1979a) separates the two species on the basis of quantitative differences in the shape of the head and T1, shape of the T1 dorsal carinae, and leg color. Slight differences in the shape of the head (Figs 18, 20: width vs. length in dorsal view 1.8 in *Opius albericus*, 2.0 in *Opius pilosicornis*) were the only features (of those listed in Fischer’s diagnosis) that we could confirm via side-by-side comparison of the two holotypes. Though the differences are subtle, we have chosen to accept the two as valid species pending collection and examination of more material to assess variation. Among the minor differences, the face appears to be more extensively shagreened in *Opius pilosicornis* but more distinctly punctate in *Opius albericus* and the metasoma is more densely setose posteriorly in *Opius albericus*. These two species are most readily separated from the others included here by the color pattern of white hind coxa, dark hind femur, and dark mesosoma.

In the original description, the locality for one of the paratypes is listed as M. Crosso but the actual locality is M. Grosso. We have seen four additional male specimens from this same locality in Mato Grosso (CNC, TAMU) but we are unable to assign them to this species with complete confidence. There are slight differences in coloration (mid and hind coxae dark brown instead of white, for example) and the propodeum of one of these specimens is distinctly granular. In one of our specimens, m-cu is postfurcal in one fore wing and weakly antefurcal in the other.

#### Opius antennatus

Fischer

http://species-id.net/wiki/Opius_antennatus

Figs 14 , 45–46

Opius antennatus Fischer, 1965a: 65–67.Holotype male in AEIC (examined).
Opius antennatus : Fischer 1971: 43 (catalog).
Opius (Merotrachys) antennatus : Fischer 1977: 655–659 (key, redescription); Fischer 1979a: 264–266 (key); Yu et al. 2005, 2012 (electronic catalogs).

##### Type locality.

USA, South Carolina, Cleveland.

##### Type material.

Holotype. Male (AEIC), first label, first line: Cleveland SC second line: VIII 2. 1952 third line: G. & L. Townes

##### Paratype.

One male (not seen), USA, South Carolina, Greenville, 12.vii.1952, G. & L. Townes.

##### Diagnosis.

Face faintly punctate, nearly smooth except shagreened adjacent eye margin. Eye in lateral view 2.5–3.0 × longer than temple; temples in dorsal view weakly receding. Male antenna with 48 flagellomeres; setae on basal flagellomeres thin, pale. Mesoscutum anteriorly with shallow but distinct declivity; notaulus extending laterally towards tegula as groove bordered by distinct supramarginal carina. Propodeum rugose to rugulose, median areola absent, median trough anteriorly shallow. Fore wing 3RSa very weakly curved, 1.35–1.4 × longer than 2RS; m-cu very weakly postfurcal. T1 sharply declivitous anteriorly, basal pit delimited posterior-medially; surface smooth to rugulose; dorsal carinae parallel-sided throughout, not sinuate, very weakly transversely carinate between dorsal carinae. T2+3 uniformly, distinctly shagreened. Head and mesosoma largely pale orange, mostly brownish orange dorsally; T1 orange, T2–4 pale medially, dark brown laterally, T5–6 dark brown; hind coxa and femur whitish; antenna without subapical pale ring; wing hyaline.

##### Remarks.

Known only from holotype and one paratype, both males. This species, described from South Carolina, USA, has the northernmost distribution of those treated here, and is the only species of the *ingenticornis* species group thus far recorded from outside of the Neotropical Region. It is also the smallest of the included species, with body length about 2.1 mm. The color pattern is distinctive, dorsally infumate on the head and mesosoma, yellow-orange below (Figs 45–46). *Opius antennatus* is closest to *Opius michaeli* in color pattern, though *Opius michaeli* has a dark mesopleuron and somewhat darker legs. The mesoscutum has a weak anterior declivity in both, but T2+3 is more distinctly shagreened in *Opius antennatus* than in *Opius michaeli*.

#### Opius curiosicornis

Fischer

http://species-id.net/wiki/Opius_curiosicornis

Figs 12 , 15 , 27 , 31 , 44

Opius curiosicornis Fischer, 1965c: 224–228.Holotype female in AEIC (examined).
Opius curiosicornis : Fischer 1965d: 420 (key); Fischer 1968a: 77–78 (key); Fischer 1971: 59 (catalog).
Opius (Merotrachys) curiosicornis : Fischer 1977: 655–657, 668–670 (key, redescription); Fischer 1979a: 264–266 (key); Yu et al. 2005, 2012 (electronic catalogs).

##### Type locality.

Peru, Avispas, near Marcapata, 30 m.

##### Type material.

Holotype. Female (AEIC), first label, first line: Avispas, Perú second line: 30m nr. Marcapata third line: Sept. 1962 fourth line: Luis Peña

##### Diagnosis.

Face faintly punctate, nearly smooth, polished throughout. Eye in lateral view 2.0–2.5 × longer than temple; temples in dorsal view weakly receding. Female antenna with 49 flagellomeres; setae on basal flagellomeres thick, dark. Mesoscutum anteriorly with shallow but distinct declivity; notaulus weakly curving laterally towards tegula, supramarginal carina weak, barely distinguishable. Propodeum weakly shagreened, largely smooth, with deep median trough divided by transverse carina into shorter anterior trough and longer, roughly rectangular posterior areola. Fore wing 3RSa straight, 1.5–1.6 × longer than 2RS; m-cu interstitial. T1 sharply declivitous anteriorly, pit delimited posterior-medially; surface weakly shagreened, mostly smooth; dorsal carinae parallel-sided for most of their length, abruptly converging near posterior margin, not sinuate, not transversely carinate between dorsal carinae. T2 mostly weakly shagreened, smoother laterally, T3 faintly shagreened to smooth, especially laterally. Ovipositor short; ovipositor sheath 0.4 × length of mesosoma. Head, body, hind coxa and femur light orange; antenna without pale subapical ring; wing darkly infumate.

##### Remarks.

Known only from the female holotype. The propodeal sculpture (Fig. 27) is similar to that of *Opius bicarinifer*, but the shape of T1 and the pronope are more typical of members of the *ingenticornis* species group. T1 is weakly excavated near the posterior margin between the dorsal and lateral carinae, resulting in a pattern that is nearly identical to that found in *Opius bicarinifer* Fischer. See remarks section under *Opius bicarinifer* below for additional information.

#### Opius filiflagellatus

Fischer

http://species-id.net/wiki/Opius_filiflagellatus

Fig. 32

Opius filicornis Fischer, 1963: 387–389.Holotype female in CAS (examined).
Opius filicornis : Fischer 1964: 3–12 (key); Fischer 1965c: 236 (comparison with *Opius ingenticornis*).
Opius filiflagellatus Fischer, 1965d: 420, 426 (key, new name); Fischer 1968a: 77–78 (key); Fischer 1971: 59 (catalog).
Opius (Merotrachys) filiflagellatus : Fischer 1977: 655–656, 673–675 (key, redescription); Fischer 1979a: 264–265 (key); Yu et al. 2005, 2012 (electronic catalogs).

##### Type locality.

Peru, Monson Valley, Tingo Maria.

##### Type material.

Holotype. Female (CAS), first label, first line: PERU: second line: Monson Valley third line: Tingo Maria fourth line: X–9–1954 second label, first line: E.I. Schlinger second line: & E. S. Ross third line: collectors

##### Diagnosis.

Face mostly faintly punctate and finely shagreened, more strongly shagreened along eye margin. Eye in lateral view 3.0–3.1 × longer than temple; temples in dorsal view very weakly receding. Female antenna with 50 flagellomeres (original description); setae on basal flagellomeres thick, dark. Mesoscutum anteriorly on nearly same plane as pronotum, without distinct anterior declivity; notaulus extending laterally towards tegula as groove bordered by distinct supramarginal carina. Propodeum coarsely, carinately rugose, with short median trough anteriorly, areola largely obscured by sculpture posteriorly. Fore wing 3RSa very weakly curved, nearly straight, 1.5 × longer than 2RS; m-cu distinctly antefurcal. T1 sharply declivitous anteriorly, pit delimited posterior-medially; surface very intensely shagreened throughout and rugulose posterior-medially, the sculpture partly obscuring dorsal carinae; dorsal carinae weakly converging, nearly parallel-sided for most of their length. T2 intensely shagreened, T3 more finely shagreened. Ovipositor broken; ovipositor sheath apparently missing (broken). Head, body, hind coxa and femur orange; antenna without pale subapical ring; wing infumate.

##### Remarks.

This species, known only from the poorly preserved holotype, was originally described as *Opius filicornis* by Fischer (1963) but the name was preoccupied by *Opius filicornis* Thomson, 1895. Fischer (1965d) subsequently renamed the species as *Opius filiflagellatus*. Both antennae are broken on the holotype, with 42 flagellomeres remaining on the longest one. The metasoma is glued to the point separately from the remainder of the specimen, and the ovipositor is broken and its full length is thus unknown. The original description states half as long as metasoma, but it is unclear if this was meant to be the total length or just the visible portion. The right fore wing is missing, as are most of the legs.

This species most closely resembles *Opius rojam* and *Opius ingenticornis* in overall appearance. The color and propodeal sculpture are the same, and *Opius filiflagellatus* similarly has T2+3 distinctly shagreened. However, the setal pattern on the basal flagellomeres would seem to remove *Opius filiflagellatus* from the subgroup of species discussed below under *Opius gabrieli*. The mesoscutum is also not quite as flattened anteriorly and the temples in dorsal view are somewhat weakly receding relative to *Opius rojam* and *Opius ingenticornis*. In existing keys to species of *Merotrachys* (Fischer 1977, 1979a), *Opius filiflagellatus* is distantly removed from *Opius ingenticornis* because of the antefurcal position of fore wing m-cu. This latter character is somewhat unreliable amongst members of the *ingenticornis* species group given variation we have seen both within series and between wings of single individuals.

#### Opius gabrieli

Fischer

http://species-id.net/wiki/Opius_gabrieli

Fig. 16

Opius gabrieli Fischer, 1968a: 77–78 (key); 84–85 (description). Holotype female in AEIC (examined).
Opius gabrieli : Fischer 1971: 68 (catalog).
Opius (Merotrachys) gabrieli : Fischer 1977: 655–657, 675–676 (key, redescription); Fischer 1978: 166 (range expansion, allotype); Fischer 1979a: 264–266 (key); Yu et al. 2005, 2012 (electronic catalogs).

##### Type locality.

Brazil, Teresópolis.

##### Type material.

Holotype. Female (AEIC), first label, first line: Teresópolis second line: III–11–66 Braz. third line: H. & M. Townes

##### Paratypes.

One female, one male (not seen), same data as holotype; one male (not seen), same data except 12.iii.1966.

##### Other specimens examined.

One female, Costa Rica, Cartago, Turrialba, 3–5.vi.1976, M. Wasbauer (TAMU).

**Other material (not examined).** Two males (one the allotype), Brazil, Carauru, iv.1972, M. Alvarenga.

##### Diagnosis.

Face finely but distinctly punctate, punctures separated by nearly 2 × their diameter, strongly shagreened adjacent eye margin, otherwise smooth between punctures. Eye in lateral view 2.0–2.5 × longer than temple; temples in dorsal view not or only weakly receding. Female antenna broken, male from original description with 50 flagellomeres, from subsequent description with 53 flagellomeres; setae on basal flagellomeres thin, pale. Mesoscutum very weakly declivitous, nearly on same plane as pronotum; notaulus extending laterally towards tegula as groove bordered by distinct supramarginal carina. Propodeum coarsely, carinately rugose, with short, deep median trough anteriorly separated from broad, irregular, ill-defined areola posteriorly. Fore wing 3RSa very weakly curved, nearly straight, 1.3–1.4 × longer than 2RS; m-cu interstitial to weakly antefurcal. T1 sharply declivitous anteriorly, pit delimited posterior-medially; surface shagreened; dorsal carinae weakly sinuate, nearly parallel-sided throughout, very weakly diverging subapically then weakly narrowing to apex, not distinctly transversely carinate between dorsal carinae. T2 mostly distinctly shagreened, smoother laterally, T3 faintly shagreened medially, smooth laterally. Ovipositor short; ovipositor sheath about 0.4 × length of mesosoma. Head, body, hind coxa and femur light orange except T3–6 infumate to completely black; wing lightly infumate.

##### Remarks.

This species was described from the female holotype plus one additional female and two male paratypes, all from the same locality in Brazil. Fischer (1978) recorded two additional males from “Carauru,” Brazil, designated one of these as the allotype, and incorrectly stated that the male was new (i.e. previously unknown). Carauru is an inadvertent misspelling of Caruaru.

*Opius gabrieli* is nearly identical to *Opius ingenticornis*, *Opius melchioricus*, and the newly described *Opius rojam*. All four species have very short ovipositors (Figs 13, 14, 16), heavily sculptured propodea (Fig. 28), thinner, pale setae on the basal flagellomeres (Fig. 39), and are predominantly orange. *Opius antennatus*, *Opius matthaei*, *Opius petri*, and *Opius raphaeli* are darker but otherwise share these features and together these eight species form a larger subgroup within the *ingenticornis* species group. *Opius gabrieli* is most readily recognized by the black apical metasomal terga relative to *Opius ingenticornis, O. melchioricus*, and *Opius rojam*. *Opius ingenticornis* and *Opius rojam* are more uniformly orange and the face is more completely shagreened than in the other two species whereas *Opius melchioricus* has the tegula black with dark transverse lines across the posterior margins of the meso- and metathorax. *Opius filiflagellatus* provides an interesting contrast since the propodeum is extensively carinately rugose and the metasoma is intensely shagreened anteriorly as in *Opius ingenticornis*, but the setal pattern on the basal flagellomeres does not match those of the subgroup delineated here.

The female specimen from Costa Rica listed above under other material examined is very similar to the holotype and we tentatively include it here. The most significant differences are in the color pattern and wing venation. The apex of the metasoma is dark in the specimen from Costa Rica, but not as contrastingly so as in the holotype. The position of the fore wing m-cu varies slightly between the two wings of the holotype, but is more distinctly postfurcal in the specimen from Costa Rica.

#### Opius ingenticornis

Fischer

http://species-id.net/wiki/Opius_ingenticornis

Figs 28 , 38

Opius ingenticornis Fischer, 1965c: 233–236. Holotype female in AEIC (examined).
Opius ingenticornis : Fischer 1965d: 420 (key); Fischer 1968a: 77–78 (key); Fischer 1971: 76 (catalog).
Opius (Merotrachys) ingenticornis : Fischer 1977: 655–657, 679–680 (key, redescription); Fischer 1979a: 264–266 (key); Yu et al. 2005, 2012 (electronic catalogs).

##### Type locality.

Peru, Quincemil, near Marcapata, 750 m.

##### Type material.

Holotype. Female (AEIC), first label, first line: Quincemil, Perú second line: 750 m nr. Marcapata third line: Nov. 10–15, 1962 fourth line: Luis Peña

##### Paratypes.

One female, one male (not seen), same data as holotype except ix.1962; one female, two males (not seen), same data except 20–30.x.1962.

##### Diagnosis.

Face shagreened throughout. Eye in lateral view 2–3 × longer than temple; temples in dorsal view not receding. Female antenna with 47–49 flagellomeres, male with up to 62 flagellomeres; setae on basal flagellomeres thin, pale. Mesoscutum anteriorly on nearly same plane as pronotum, without distinct anterior declivity; notaulus extending laterally towards tegula as groove bordered by distinct supramarginal carina. Propodeum coarsely, carinately rugose and shagreened, with short median trough anteriorly, areola largely obscured by sculpture posteriorly. Fore wing 3RSa very weakly curved, nearly straight, 1.25 × longer than 2RS; m-cu interstitial to very weakly postfurcal. T1 sharply declivitous anteriorly, pit delimited posterior-medially; surface very intensely shagreened; dorsal carinae distinctly elevated, nearly parallel-sided throughout, weakly converging near apex, not sinuate, not obviously transversely carinate between dorsal carinae. T2+T3 distinctly shagreened. Ovipositor short; ovipositor sheath about 0.3–0.4 × length of mesosoma. Head, body, hind coxa and femur orange; antenna without pale subapical ring; wing weakly infumate.

##### Remarks.

This species was originally described from the female holotype plus two female and three male paratypes, all from Peru. *Opius ingenticornis* is characterized by the extensively shagreened facial sculpture and pale body. This species is very similar to *Opius rojam*, newly described above, based on coloration, relatively small second submarginal cell of the fore wing, and propodeal sculpture. *Opius ingenticornis* is somewhat smaller, with T1 more intensively shagreened (Fig. 38) whereas *Opius rojam* is more rugose (Fig. 37). See remarks under *Opius rojam*, *Opius filiflagellatus*, and *Opius gabrieli* for additional characteristics and diagnostic features relative to other members of this species group.

#### Opius macrocornis

Fischer

http://species-id.net/wiki/Opius_macrocornis

Fig. 26

Opius macrocornis Fischer, 1965b: 298–300. Holotype male in AEIC (examined).
Opius macrocornis : Fischer 1965d: 419 (key); Fischer 1969: 162–163 (key); Fischer 1971: 84 (catalog).
Opius (Pendopius) macrocornis : Fischer 1977: 714–715, 727–728 (key, redescription); Fischer 1979b: 484–486, 495 (key); Yu et al. 2005, 2012 (electronic catalogs).

##### Type locality.

Peru, Quincemil, near Marcapata, 750 m.

##### Type material.

Holotype. Male (AEIC), first label, first line: Quincemil, Perú second line: 750 m nr. Marcapata third line: Nov. 10-15, 1962 fourth line: Luis Peña Sept.

##### Diagnosis.

Face very faintly punctate, otherwise smooth, polished throughout. Eye in lateral view 2.4–2.6 × longer than temple; temples in dorsal view not or only weakly receding. Male antenna with 45 flagellomeres; setae on basal flagellomeres thick, dark. Mesoscutum with weak declivity; supramarginal carina absent or apparently so. Propodeum smooth, polished with shallow median trough anteriorly continuous with broader, weakly defined areola posteriorly. Fore wing 3RSa straight, about 1.6 × longer than 2RS; m-cu postfurcal. T1 evenly curving into basal pit anteriorly, not distinctly declivitous, pit well-defined, delimited posterior-medially; surface smooth, polished; dorsal carinae parallel-sided for most of their length, distinctly converging near posterior margin, not sinuate, not transversely carinate between dorsal carinae. T2+T3 smooth, polished. Head, body, hind coxa and femur pale orange; antenna without pale subapical ring; wing darkly infumate.

##### Remarks.

This species is known only from the male holotype and is very similar to *Opius nimifactus*, as noted by Fischer (1979b). Both species are characterized by greatly reduced propodeal sculpture (Figs 25–26), relatively smooth T1, and absence of any shagreening on T2. T1 anteriorly is more gradually sloping in *Opius macrocornis*, and *Opius macrocornis* is more uniformly pale orange: lacking the black tegula and dark margins of the mesoscutum that characterize *Opius nimifactus*. There is a patch of sculpture between the notaulus and the anterior-lateral margin of the mesoscutum in *Opius nimifactus* but this area is largely smooth in *Opius macrocornis*. The mesoscutum is also weakly declivitous in *Opius macrocornis* but flatter in *Opius nimifactus*. Fischer (1979b) provides additional comparison of the two species. Both of these species were placed in the subgenus *Pendopius* by Fischer (1977, 1979b) because of the absence of sculpture on T2. The shagreened sculpture on the metasoma appears to vary intraspecifically in opiines when there is sufficient material for comparison, and is often extremely weak in some of the species of the *ingenticornis* species group. We therefore do not consider the sculpture pattern alone to be adequate for characterizing subgenera or species groups, and treat it as variably present or absent in the *ingenticornis* species group. Both *Opius macrocornis* and *Opius nimifactus* fall within our concept of the *ingenticornis* species group, resembling species with relatively reduced sculpture and darker, thicker flagellar setae such as *Opius curiosicornis*.

#### Opius matthaei

Fischer

http://species-id.net/wiki/Opius_matthaei

Figs 1 , 30 , 34

Opius matthaei Fischer, 1968a: 77–78 (key); 90–92 (description). Holotype female in AEIC (examined).
Opius matthaei : Fischer 1971: 86 (catalog).
Opius (Merotrachys) matthaei : Fischer 1977: 655–656, 685–687 (key, redescription); Fischer 1979a: 264 (key); Yu et al. 2005, 2012 (electronic catalogs).

##### Type locality.

Brazil, Campina Grande, near Curitiba.

##### Type material.

Holotype. Female (AEIC), first label, first line: Campina Grande second line: nr. Curitiba third line: II–17–66 Brazil fourth line: H.&M. Townes

##### Diagnosis.

Face finely granular or coarsely shagreened throughout. Eye in lateral view 2.4–2.7 × longer than temple; temples in dorsal view not receding. Female antenna with 57 flagellomeres; setae on basal flagellomeres thin, pale. Mesoscutum anteriorly on nearly same plane as pronotum, without distinct anterior declivity; notaulus extending laterally towards tegula as groove bordered by distinct supramarginal carina, base of notaulus weakly rugulose, thus appearing to extend to anterior margin of mesoscutum. Propodeum rugulose to finely granular with shallow median trough anteriorly, areola obscured by sculpture posteriorly. Fore wing 3RSa weakly curved, 1.3 × longer than 2RS; m-cu weakly antefurcal. T1 sharply declivitous anteriorly, pit delimited posterior-medially; surface rugulose between dorsal carinae, shagreened laterally; dorsal carinae weakly sinuate, broadening subapically, narrowing apically. T2 distinctly shagreened, T3 more weakly so, becoming smooth, polished laterally. Ovipositor short; ovipositor sheath about 0.3–0.4 × length of mesosoma. Head, propodeum, T1, T4–T6 dark reddish brown to dark brown; mesosoma mottled dark orange to dark reddish brown; T2+T3 reddish brown; hind coxa and femur dark yellow; antenna without subapical pale ring; wing lightly infumate, nearly hyaline.

##### Remarks.

This species is known only from the female holotype. It is a relatively dark species, most closely resembling *Opius albericus* and *Opius pilosicornis* in that regard, but the legs are more uniformly yellow in *Opius matthaei* (Figs 17, 19, 34). In keys to species of the subgenus *Merotrachys* (Fischer 1977, 1979a), *Opius matthaei* is widely separated from *Opius albericus* and *Opius pilosicornis* because of slight differences in the position of fore wing m-cu (antefurcal as in Fig. 30, but only weakly so). *Opius matthaei* is most readily characterized by the densely granular facial sculpture (Fig. 1).

**Figures 33–36.**
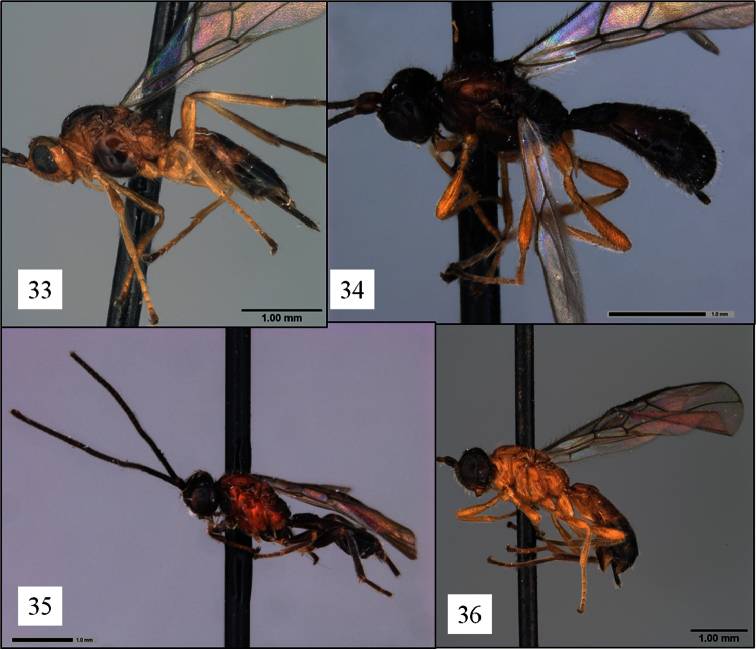
*Opius* spp. holotypes, habitus. **33** *Opius michaeli* Fischer **34** *Opius matthaei* Fischer **35** *Opius petri* Fischer **36** *Opius raphaeli* Fischer.

**Figures 37–40.**
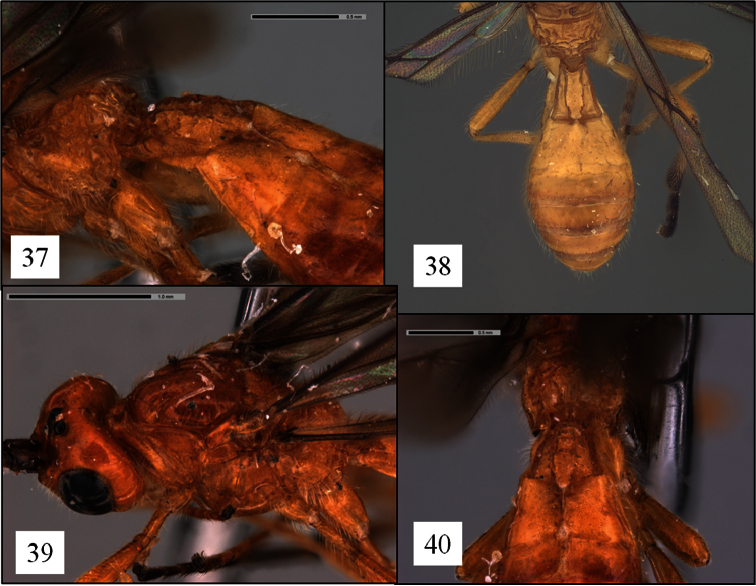
*Opius* spp. holotypes. **37** *Opius rojam* Daniels & Wharton sp. n., T1 dorsal-lateral view **38** *Opius ingenticornis* Fischer, T1 dorsal view **39** *Opius rojam*, mesosoma oblique view **40** *Opius rojam*, T2+3 showing deformity.

#### Opius melchioricus

Fischer

http://species-id.net/wiki/Opius_melchioricus

Figs 3 , 5

Opius (Merotrachys) melchioricus Fischer, 1979a: 264–266 (key); 271–273 (description). Holotype male in AEIC (examined).
Opius (Merotrachys) melchioricus : Yu et al. 2005, 2012 (electronic catalogs).

##### Type locality.

Brazil, Guanabara, Reprêsa Rio Grande.

##### Type material.

Holotype. Male (AEIC), first label, first line: ReprêsaRioGrande second line: Guanabara, Brazil third line: January, 1968 Brazil fourth line: M. Alvarenga.

##### Other specimens examined.

One female, same data as holotype (TAMU).

##### Diagnosis.

Face finely but distinctly punctate, punctures separated by nearly 2 × their diameter, strongly shagreened adjacent eye margin, otherwise smooth between punctures. Eye in lateral view roughly 2.5 (male) and 3.0 (female) × longer than temple; temples in dorsal view not or only weakly receding. Female antenna with 53 flagellomeres, male holotype with 56–57 flagellomeres; setae on basal flagellomeres thin, pale. Mesoscutum anteriorly on nearly same plane as pronotum, without distinct anterior declivity; notaulus extending laterally towards tegula as groove bordered by distinct supramarginal carina. Propodeum coarsely carinately rugose with deep median trough anteriorly separated by carina from broad median, roughly pentagonal to elliptical areola posteriorly, areola carinately sculptured medially. Fore wing 3RSa straight, 1.3 × longer than 2RS; m-cu postfurcal. T1 sharply declivitous anteriorly, pit delimited posterior-medially; surface carinately rugose medially, rugulose laterally; dorsal carinae distinctly elevated, nearly parallel-sided throughout, weakly converging posteriorly, not sinuate, transversely carinate between dorsal carinae. T2 and T3 distinctly shagreened. Ovipositor short; ovipositor sheath about 0.3–0.4 × length of mesosoma. Head, body, hind coxa and femur orange to pale orange; tegula, posterior margins of mesonotum, metanotum, and T3–6 dark reddish brown, T6 more uniformly weakly infumate; antenna without pale subapical ring; wing infumate in male, more nearly hyaline in female.

##### Remarks.

Previously known only from the male holotype. Female characters are based on a specimen collected at the type locality in Brazil (TAMU). The female differs from the holotype primarily in wing venation, with 3RSa about 1.5 × longer than 2RS and the wing is more nearly hyaline. Otherwise, body coloration and sculpture are the same. This species is characterized by the dark transverse markings on the posterior margins of the meso- and metathorax. It is most similar to *Opius rojam*, *Opius gabrieli* and *Opius ingenticornis*. For a detailed comparison, see remarks section under *Opius gabrieli*.

#### Opius michaeli

Fischer

http://species-id.net/wiki/Opius_michaeli

Figs 22, 24 , 33

Opius michaeli Fischer, 1968a: 77–78 (key); 92–95 (description). Holotype female in AEIC (examined).
Opius michaeli : Fischer 1971: 87 (catalog).
Opius (Merotrachys) michaeli : Fischer 1977: 655–657, 687–689 (key, redescription); Fischer 1979a: 264–266 (key); Fischer 1983b: 83 (diagnosis in couplet of key); Yu et al. 2005, 2012 (electronic catalogs).

##### Type locality.

Brazil, Teresópolis.

##### Type material.

Holotype. Female (AEIC), first label, first line: Teresópolis second line: III–12–66 Braz. third line: H. & M. Townes

##### Paratypes.

One male (not seen), same data as holotype; one female (not seen), Brazil, Campina Grande, near Curitiba, 22.ii.1966, H. & M. Townes.

##### Diagnosis.

Face faintly punctate, otherwise smooth. Eye in lateral view about 4 × longer than temple; temples in dorsal view weakly receding. Female antenna with 46–48 flagellomeres, male with 45 flagellomeres; setae on basal flagellomeres thin, pale. Mesoscutum anteriorly with shallow but distinct declivity; notaulus extending laterally towards tegula as groove bordered by distinct supramarginal carina. Propodeum coarsely carinately rugose on posterior 0.6, nearly smooth anteriorly, with deep median trough anteriorly, areola obscured by sculpture posteriorly. Fore wing 3RSa straight or nearly so, 1.4 × longer than 2RS; m-cu postfurcal. T1 sharply declivitous anteriorly, pit delimited posterior-medially; surface rugose to rugulose; dorsal carinae weakly sinuate, nearly parallel-sided, broadening subapically, narrowing apically, weakly transversely carinate between dorsal carinae. T2 faintly shagreened, T3 mostly smooth. Ovipositor moderately short, but longer than most other species in this species group; ovipositor sheath about 0.5–0.6 × length of mesosoma. Head, prothorax, propodeum, and T1 yellow-orange; meso- and metathorax, T2 and T5–6 mostly brown, T3–4 yellow-brown; hind coxa and femur yellow; antenna without pale subapical ring; wing weakly infumate.

##### Remarks.

This species was originally described from the female holotype plus a male and a female paratype. The female is readily distinguished from all others included here in the *ingenticornis* species group by the slightly longer ovipositor and mottled color pattern (Fig. 33). The species with longer ovipositors treated below have all been excluded from this species group on the basis of other features and thus, where known, members of the *ingenticornis* species group all have relatively short ovipositors, with the ovipositor sheath distinctly shorter than the mesosoma. The propodeum of *Opius michaeli* is generally similar in sculpture to those species in the subgroup discussed under the remarks section for *Opius gabrieli*, but is nearly smooth anteriorly. Fischer (1983b) compared *Opius michaeli* to *Opius monsonicus* from Peru and both have similarly long ovipositors. Although *Opius monsonicus* has antennae that are very long as in members of the *ingenticornis* species group, we have excluded this species primarily on the basis of the propodeum, which is described as having a basal keel or midridge.

#### Opius nimifactus

Fischer

http://species-id.net/wiki/Opius_nimifactus

Figs 25 , 42

Opius (Pendopius) nimifactus Fischer, 1979b: 484–486 (key); 493–495 (description). Holotype female in AEIC (examined).
Opius (Pendopius) nimifactus : Yu et al. 2005, 2012 (electronic catalogs).

##### Type locality.

Brazil, Caruaru.

##### Type material.

Holotype. Female (AEIC), first label, first line: Caruaru, Brazil second line: May 1972 900m. third line: J. Lima

##### Paratypes.

Two females (not seen), same data as holotype except vi.1972; five females, three males (examined), same locality but iv.1972, M. Alvarenga.

##### Diagnosis.

Face distinctly punctate, punctures separated by about 2 × their diameter, strongly shagreened adjacent eye margin, otherwise largely smooth between punctures. Eye in lateral view 2.0–2.5 × longer than temple; temples in dorsal view not receding. Female antenna with 59–61 flagellomeres, male with 56–60 flagellomeres; setae on basal flagellomeres thick, dark. Mesoscutum anteriorly on nearly same plane as pronotum, without distinct anterior declivity; notaulus extending laterally towards tegula as groove bordered by distinct supramarginal carina, also extending to anterior margin of mesoscutum as a weak groove. Propodeum nearly smooth: finely rugulose punctate with shallow median trough anteriorly, becoming flat, without areola posteriorly. Fore wing 3RSa curved, about 1.4–1.7 × longer than 2RS; m-cu postfurcal. T1 declivitous anteriorly at about a 45 degree angle, pit delimited posterior-medially; surface smooth to weakly shagreened; dorsal carinae very weakly sinuate, nearly parallel-sided, weakly broadening subapically, weakly narrowing apically, not transversely carinate between dorsal carinae. T2 and T3 smooth, polished. Ovipositor short; ovipositor sheath about 0.3–0.4 × length of mesosoma. Head, body, hind coxa and femur pale orange; tegula and lateral margin of mesonotum dark brown to black, T5 with dark maculae laterally, T6 uniformly dark brown; antenna without pale subapical ring; wing darkly infumate.

##### Remarks.

This species was described from the female holotype plus 7 additional female and 3 male paratypes, all from same locality. *Opius nimifactus* is similar in many respects to *Opius macrocornis*, as detailed in the remarks section under that species. *Opius macrocornis* is more uniformly pale orange: lacking the black tegula and dark margins of the mesoscutum that characterize *Opius nimifactus*.

#### Opius petri

Fischer

http://species-id.net/wiki/Opius_petri

Fig. 35

Opius petri Fischer, 1968a: 77 (key); 95–98 (description). Holotype female in AEIC (examined).
Opius petri : Fischer 1971: 98 (catalog).
Opius (Merotrachys) petri : Fischer 1977: 655–656, 698–700 (key, redescription); Fischer 1979a: 264–266 (key); Yu et al. 2005, 2012 (electronic catalogs).

##### Type locality.

Suriname, near Paramaribo.

##### Type material.

Holotype. Female (AEIC), first label, first line: nr. Paramaribo second line: Surinam second label, first line: XII.7–13.63 second line: D.C. Geijskes

##### Diagnosis.

Face weakly shagreened medially, strongly shagreened adjacent eye margin, with scattered large punctures. Eye in lateral view 2.0–2.5 × longer than temple; temples in dorsal view not receding. Female antenna broken; setae on basal flagellomeres thin, pale. Mesoscutum anteriorly gradually merging with plane of pronotum, with weakly elevated, indistinct anterior declivity; notaulus extending laterally towards tegula as groove bordered by distinct supramarginal carina. Propodeum rugose with heavily sculptured median trough anteriorly, areola obscured by sculpture posteriorly. Fore wing 3RSa weakly curved, about 1.2 × longer than 2RS; m-cu postfurcal. T1 sharply declivitous anteriorly, pit delimited posterior-medially; surface weakly rugulose and shagreened; dorsal carinae irregularly sinuate, broadest posteriorly; weakly transversely carinate between dorsal carinae. T2 and T3 shagreened. Ovipositor short; ovipositor sheath about 0.3–0.4 × length of mesosoma. Head, prothorax, tegula, most of propodeum, T1, T3–T6, hind coxa and femur dark reddish brown; mesosoma otherwise mostly dark orange, T2 medially orange-brown with narrow, dark brown lateral margins; wing lightly infumate.

##### Remarks.

This species is known only from the female holotype and is readily recognizable by the distinctive color pattern of dark head and legs and mostly dark orange mesosoma (Fig. 35). T1 is also a bit shorter than in other dark species such as *Opius albericus* and *Opius pilosicornis*.

In the original description, the collector’s name is incorrectly spelled Geijkes.

#### Opius pilosicornis

Fischer

http://species-id.net/wiki/Opius_pilosicornis

Figs 19, 20

Opius pilosicornis Fischer, 1965c: 239–242. Holotype female in AEIC (examined).
Opius pilosicornis : Fischer 1965d: 420 (key); Fischer 1968a: 77–78 (key); Fischer 1971: 76 (catalog).
Opius (Merotrachys) pilosicornis : Fischer 1977: 655–657, 700–701 (key, redescription); Fischer 1978: 166 (range extension); Fischer 1979a: 264–267 (key); Yu et al. 2005, 2012 (electronic catalogs).

##### Type locality.

Peru, Quincemil, near Marcapata, 750 m.

##### Type material.

Holotype. Female (AEIC), first label, first line: Quincemil, Perú second line: 750 m nr Marcapata third line: Nov. 10–15, 1962 fourth line: Luis Peña

##### Paratypes.

Two females (not seen), same data as holotype; one female, same data except ix.1962.

##### Other material 

**(not examined).** One female, one male, Brazil, Para, Jacareacanga, x.68, M. Alvarenga.

##### Diagnosis.

Face distinctly punctate, punctures separated by about 1 × their diameter, strongly shagreened adjacent eye margin, otherwise mostly weakly shagreened between punctures. Eye in lateral view 2.0–2.5 × longer than temple; temples in dorsal view not receding. Antenna of female with 56 flagellomeres, allotype with 54 flagellomeres; setae on basal flagellomeres thick, dark. Mesoscutum anteriorly on nearly same plane as pronotum, without distinct anterior declivity; notaulus extending laterally towards tegula as groove bordered by distinct supramarginal carina. Propodeum coarsely rugose, median areola absent, median trough anteriorly deep, short, distinct. Fore wing 3RSa straight, about 1.6 × longer than 2RS; m-cu postfurcal. T1 declivitous anteriorly at about a 45 degree angle, basal pit delimited posterior-medially; surface shagreened throughout; dorsal carinae weakly elevated, sinuate, widest subapically, narrowing apically, without obvious transverse carinae between dorsal carinae. T2 uniformly, distinctly shagreened; T3 mostly weakly shagreened, smoother and very finely punctate laterally. Ovipositor short, barely protruding; ovipositor sheath roughly 0.4 × length of mesosoma. Head, mesosoma, T1, T3–T6 dark reddish brown to dark brown; T2 white with narrow, dark brown lateral margins; hind coxa white; hind femur almost completely dark reddish brown; antenna without subapical pale ring; wing lightly infumate.

##### Remarks.

This species was described from the holotype female and three additional paratype females, all from Peru. Fischer (1978) subsequently recorded a male and a female from the state of Pará in Brazil. This species is nearly identical to *Opius albericus* (see remarks above under that species), differing only in minor details, most notably in the relative size of the eye.

#### Opius raphaeli

Fischer

http://species-id.net/wiki/Opius_raphaeli

Figs 2 , 11 , 36

Opius raphaeli Fischer, 1968a: 77–78 (key); 98–101 (description). Holotype female in AEIC (examined).
Opius raphaeli : Fischer 1971: 104 (catalog).
Opius (Merotrachys) raphaeli : Fischer 1977: 655–657, 702–704 (key, redescription); Fischer 1979a: 264–266 (key); Yu et al. 2005, 2012 (electronic catalogs).

##### Type locality.

Argentina, Horco Molle, near Tucumán.

##### Type material.

Holotype. Female (AEIC), first label, first line: Horco Molle second line: nr. Tucumán third line: I–18–66 Arg. fourth line: H. & M. Townes

##### Diagnosis.

Face very deeply, distinctly punctate, punctures separated by 1–2 × their diameter, strongly shagreened adjacent eye margin, otherwise very weakly shagreened to smooth between punctures. Eye in lateral view about 1.5–1.7 × longer than temple; temples in dorsal view receding. Female antenna with 53 flagellomeres; setae on basal flagellomeres thin, pale. Mesoscutum anteriorly on nearly same plane as pronotum, without distinct anterior declivity; notaulus appears longer than in other species with supramarginal carina barely indicated: appearance of both may have been altered by the pin through the mesoscutum. Propodeum rugose with sculptured median trough anteriorly separated by carina posteriorly from irregularly pentagonal areola. Fore wing 3RSa weakly curved, about 1.25–1.35 × longer than 2RS; m-cu postfurcal. T1 sharply declivitous anteriorly, pit delimited posterior-medially; surface largely smooth; dorsal carinae weakly sinuate, nearly parallel-sided, broadening subapically, narrowing apically, with a few, weak transverse carinae between dorsal carinae. T2 very faintly shagreened, T3 smooth, polished. Ovipositor short; ovipositor sheath about 0.3–0.4 × length of mesosoma. Head, posterior margin of T3, and all of T4–6 dark brown to black. Mesosoma, hind coxa, and hind femur orange; T1, T2, most of T3 a little darker: reddish orange; antenna without subapical pale ring; wing lightly infumate.

##### Remarks.

Known only from the female holotype. The granular-punctate sculpture of the clypeus (Fig. 2) is distinctive relative to other species treated here. The color pattern (Fig. 36) is also unique.

### The *ingenticornis* species group, excluded species

#### Opius bicarinifer

Fischer

http://species-id.net/wiki/Opius_bicarinifer

Figs 10 , 23

Opius (Merotrachys) bicarinifer Fischer, 1979a: 264–265 (key); 269–271 (description). Holotype male in AEIC (examined).
Opius (Merotrachys) bicarinifer : Yu et al. 2005, 2012 (electronic catalogs).

##### Type locality.

Brazil, Rondonia, Vilhena.

##### Type material.

Holotype. Female (AEIC), first label, first line: Vilhena, Rond. second line: XI. ’73 Brazil third line: M. Alvarenga

##### Diagnosis.

Face polished throughout, faintly punctate, nearly smooth. Eye in lateral view 4.0–4.5 × longer than temple; temples in dorsal view distinctly receding. Antenna broken; setae on basal flagellomeres thick, dark. Mesoscutum anteriorly with weak but distinct declivity; notaulus shallowly curving laterally towards tegula, supramarginal carina weak, barely distinguishable. Propodeum largely smooth, with deep median trough from base to apex, not broadened posteriorly into distinct areola. Fore wing 3RSa straight, 1.5–1.6 × longer than 2RS; m-cu antefurcal. T1 evenly curving into basal pit anteriorly, not distinctly declivitous, pit not delimited posterior-medially; surface weakly shagreened, nearly smooth throughout; dorsal carinae weakly sinuate, parallel-sided for most of their length, weakly broadening subapically, abruptly converging near posterior margin, not transversely carinate between dorsal carinae. T2 uniformly, distinctly shagreened, T3 more weakly so, especially laterally. Ovipositor short; ovipositor sheath 0.4 × length of mesosoma. Head, body, hind coxa and femur light orange; wing darkly infumate.

##### Remarks.

Known only from the female holotype. This species is tentatively excluded from the *ingenticornis* species group primarily because of the evenly curved anterior slope of T1 (Fig. 10) and the poorly developed malar sulcus. Since the antenna is broken, length cannot be used to assist in placement of this species. *Opius bicarinifer* is most similar to *Opius curiosicornis* because of similarities in the propodeal sculpture and both have the same color pattern. They differ primarily in shape of the head and the anterior slope of T1. The temples in dorsal view are noticeably receding in *Opius bicarinifer*, another feature not found in typical members of the *ingenticornis* species group.

#### Opius duplocarinatus

Fischer

http://species-id.net/wiki/Opius_duplocarinatus

Figs 41 , 47

Opius duplocarinatus Fischer, 1965b: 286–289. Holotype female in AEIC (examined).
Opius duplocarinatus : Fischer 1965d: 419 (key); Fischer, 1968b: 463–464 (key); Fischer 1971: 63 (catalog).
Opius (Pendopius) duplocarinatus : Fischer 1977: 714, 721–723 (key, redescription); Fischer 1979b: 484–485 (key); Yu et al. 2005, 2012 (electronic catalogs).

##### Type locality.

Peru, Avispas, near Marcapata, 30 m.

##### Type material.

Holotype. Female (AEIC), first label, first line: Avispas, Perú second line: 30m nr. Marcapata third line: Oct. 1–15, 1962 fourth line: Luis Peña

##### Diagnosis.

Face distinctly punctate, punctures separated by about 1 × their diameter laterally, more closely spaced medially, nearly smooth between punctures. Eye in lateral view about 4.5 × longer than temple; temples in dorsal view strongly receding. Female antenna with 31 flagellomeres; setae on basal flagellomeres short, moderately thick, dark. Mesoscutum anteriorly with distinct declivity; notaulus extending laterally towards tegula as groove bordered by distinct supramarginal carina. Propodeum mostly rugulose, especially anteriorly, with narrow, shallow median trough anteriorly confluent with large, broad, roughly pentagonal areola posteriorly. Fore wing 3RSa weakly curved, 1.4–1.5 × longer than 2RS; m-cu postfurcal. T1 evenly curving into basal pit anteriorly, not distinctly declivitous, pit not delimited posterior-medially; surface rugulose throughout; dorsal carinae parallel-sided for most of their length, abruptly converging near posterior margin, not or only very weakly sinuate, rugulose but not transversely carinate between dorsal carinae. T2 and T3 smooth, polished throughout. Ovipositor long; ovipositor sheath about 1.5 × longer than mesosoma. Head, body, hind coxa and femur light orange; antenna with whitish subapical ring; wing darkly infumate.

##### Remarks.

This species is known only from the female holotype and is most similar to *Opius marci*, treated below. Both species have a pale subapical ring on the antenna (Fig. 47) whereas the flagellum is uniformly dark in all other species treated here. The setal pattern on the basal flagellomeres of these two species is also similar, with the setae shorter and not quite as thick as in species such as *Opius albericus*, but thicker and darker than in species such as *Opius matthaei*. Both species also have a relatively long ovipositor and relatively short antenna (with 29–31 flagellomeres). Exclusion of these two species from the *ingenticornis* species group is based primarily on the short antennae and the T1 profiles that are concave and gradually sloping anteriorly, and secondarily on the smaller pronope. Although *Opius duplocarinatus* and *Opius marci* are nearly identical, they have been placed in different subgenera (Fischer 1977, 1979a, b) because *Opius marci* has very faintly shagreened sculpture on T2 and T2 sculpture is lacking in *Opius duplocarinatus*. There are also minor differences in the propodeum, with the areola more discrete in *Opius marci*, and *Opius duplocarinatus* has a distinct (though unsculptured) precoxal sulcus.

**Figures 41–44.**
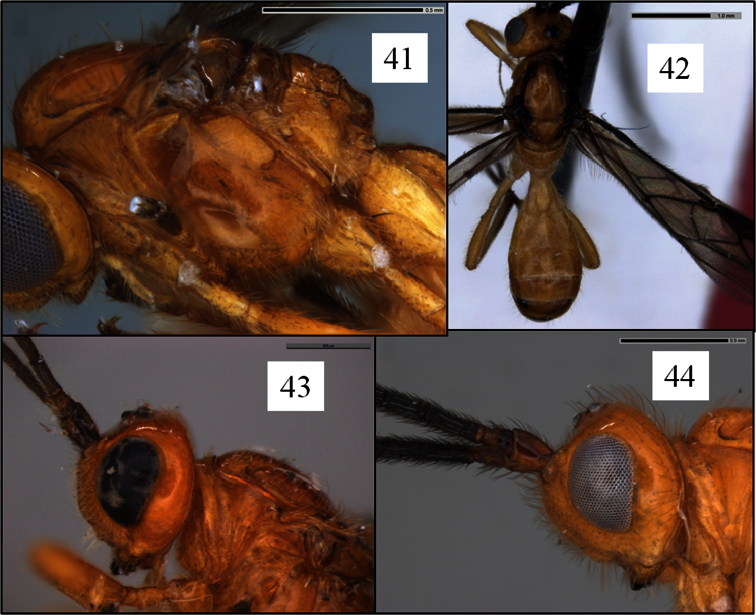
*Opius* spp. **41** *Opius duplocarinatus* Fischer, holotype, mesoscutum showing distinct anterior declivity **42** *Opius nimifactus* Fischer, paratype, dorsal view showing color pattern **43** *Opius rojam* Daniels & Wharton sp. n., holotype, head lateral view showing setal pattern on basal flagellomeres **44** *Opius curiosicornis* Fischer, holotype, head lateral view showing setal pattern on basal flagellomeres.

**Figures 45–48.**
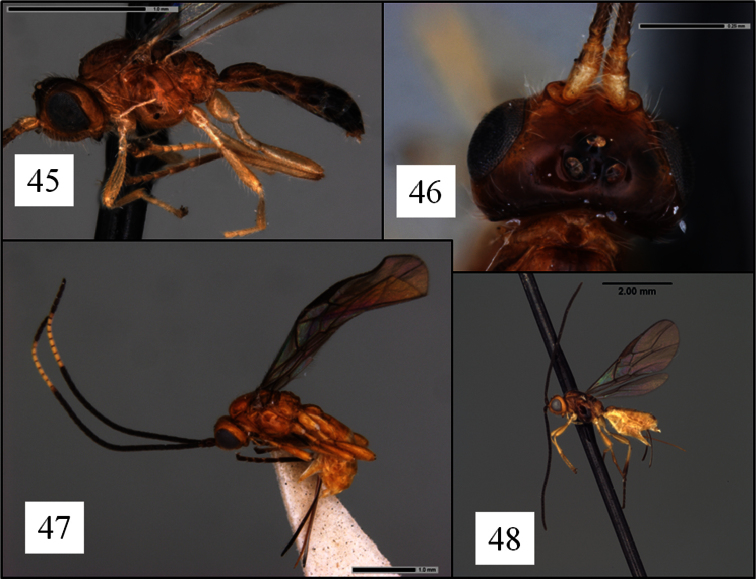
*Opius* spp. holotypes. **45** *Opius antennatus* Fischer, habitus **46** *Opius antennatus* head dorsal view **47** *Opius duplocarinatus* Fischer, habitus **48** *Opius simplicornis* Fischer, habitus.

#### Opius marci

Fischer

http://species-id.net/wiki/Opius_marci

Opius marci Fischer, 1968a: 77–78 (key); 87–90 (description). Holotype female in AEIC (examined).
Opius marci : Fischer 1971: 86 (catalog).
Opius (Merotrachys) marci : Fischer 1977: 655–657, 682–683 (key, redescription); Fischer 1979a: 264–266 (key); Yu et al. 2005, 2012 (electronic catalogs).

##### Type locality.

Peru, Loromayo.

##### Type material.

Holotype. Female (AEIC), first label, first line: Loromayo, Perú second line: IX. 4–10. 62 third line: Luis Peña

##### Diagnosis.

Face distinctly punctate, punctures separated by 1–2 × their diameter, smooth between punctures. Eye in lateral view about 3.8–4.0 × longer than temple; temples in dorsal view receding. Female antenna with 29 flagellomeres; setae on basal flagellomeres short, thick, dark. Mesoscutum anteriorly with distinct declivity; notaulus extending laterally towards tegula as groove bordered by distinct supramarginal carina. Propodeum weakly rugulose to smooth with anterior median trough separated from well-defined, discretely margined, pentagonal areola posteriorly. Fore wing 3RSa weakly curved, 1.5 × longer than 2RS; m-cu postfurcal. T1 evenly curving into basal pit anteriorly, not distinctly declivitous, pit not delimited posterior-medially; surface rugulose, at least medially; dorsal carinae parallel-sided throughout, not sinuate, transversely carinate between dorsal carinae. T2 very faintly shagreened medially, mostly smooth, polished; T3 smooth, polished. Ovipositor long; ovipositor sheath about 1.3–1.4 × longer than mesosoma. Head, body, hind coxa and femur pale orange; antenna with pale subapical ring; wing infumate.

##### Remarks.

This species is known only from the female holotype. It is nearly identical to *Opius duplocarinatus*, but has a somewhat more discrete propodeal areola. See remarks section under *Opius duplocarinatus* for additional comparisons and rationale for exclusion from the *ingenticornis* species group.

#### Opius simplicornis

Fischer

http://species-id.net/wiki/Opius_simplicornis

Fig. 48

Opius simplicornis Fischer, 1968b: 463–464 (key), 477–479 (description). Holotype female in AEIC (examined).
Opius simplicornis : Fischer 1971: 111 (catalog).
Opius (Pendopius) simplicornis : Fischer 1977: 714, 736–748 (key, redescription); Fischer 1979b: 484–485 (key); Fischer 1983a: 92; Yu et al. 2005, 2012 (electronic catalogs).

##### Type locality.

Argentina, Horco Molle, near Tucumán.

##### Type material.

Holotype. Female (AEIC), first label, first line: Horco Molle second line: nr. Tucumán third line: III.27–31.66 Arg. fourth line: Lionel Stange

##### Diagnosis.

Face finely but distinctly punctate, punctures separated by nearly 2 × their diameter, otherwise smooth between punctures. Eye in lateral view about 2.6–2.9 × longer than temple; temples in dorsal view strongly receding. Female antenna with 30 flagellomeres; setae on basal flagellomeres thick, dark. Mesoscutum anteriorly on nearly same plane as pronotum, without distinct anterior declivity; notaulus extending laterally towards tegula as groove bordered by distinct supramarginal carina. Propodeum rugulose with median trough anteriorly separated by carina posteriorly from fairly well-defined pentagonal areola. Fore wing 3RSa straight, about 1.6 × longer than 2RS; m-cu postfurcal. T1 evenly curving into basal pit anteriorly, not distinctly declivitous, pit not delimited posterior-medially; surface largely smooth; dorsal carinae weakly sinuate, broadening subapically, narrowing apically, not obviously transversely carinate between dorsal carinae. T2 and T3 smooth, polished. Ovipositor long; ovipositor sheath about 1.0–1.1 × longer than mesosoma. Head, propodeum, T1 pale orange; mesosoma mottled dark orange, and brown; T2–5, hind coxa, and hind femur yellow; T6 at least partly dark brown; antenna without subapical pale ring; wing lightly infumate.

**Remarks.** This species is known only from the female holotype. The holotype matches the original written description, but the figure (Fischer 1968b, fig. 7) is not of this species because it shows a very short ovipositor. The written description indicates a much longer ovipositor, as evident in the holotype (Fig. 48).

Fischer (1979b, p. 493) compared *Opius simplicornis* to *Opius caudisignatus* Fischer and later (Fischer 1983a) to *Opius vinoanus* Fischer. The former has a much longer ovipositor and the latter a more heavily sculptured T1 relative to *Opius simplicornis*. The absence of a distinct anterior declivity on the mesoscutum suggests a relationship to members of the *ingenticornis* species group, but the antenna is shorter, with significantly fewer flagellomeres and T1 lacks the steep anterior declivity typical of nearly all members of this species group.

## Supplementary Material

XML Treatment for Opius rojam

XML Treatment for Opius albericus

XML Treatment for Opius antennatus

XML Treatment for Opius curiosicornis

XML Treatment for Opius filiflagellatus

XML Treatment for Opius gabrieli

XML Treatment for Opius ingenticornis

XML Treatment for Opius macrocornis

XML Treatment for Opius matthaei

XML Treatment for Opius melchioricus

XML Treatment for Opius michaeli

XML Treatment for Opius nimifactus

XML Treatment for Opius petri

XML Treatment for Opius pilosicornis

XML Treatment for Opius raphaeli

XML Treatment for Opius bicarinifer

XML Treatment for Opius duplocarinatus

XML Treatment for Opius marci

XML Treatment for Opius simplicornis
